# Capturing Enzyme-Loaded Diblock Copolymer Vesicles Using an Aldehyde-Functionalized Hydrophilic Polymer Brush

**DOI:** 10.1021/acs.langmuir.4c01561

**Published:** 2024-06-27

**Authors:** Georgios Karchilakis, Spyridon Varlas, Edwin C. Johnson, Oleta Norvilaite, Matthew A. H. Farmer, George Sanderson, Graham J. Leggett, Steven P. Armes

**Affiliations:** † Dainton Building, Department of Chemistry, The University of Sheffield, Brook Hill, Sheffield, South Yorkshire S3 7HF, U.K.; ‡ GEO Specialty Chemicals, Hythe, Southampton, Hampshire SO45 3ZG, U.K.

## Abstract

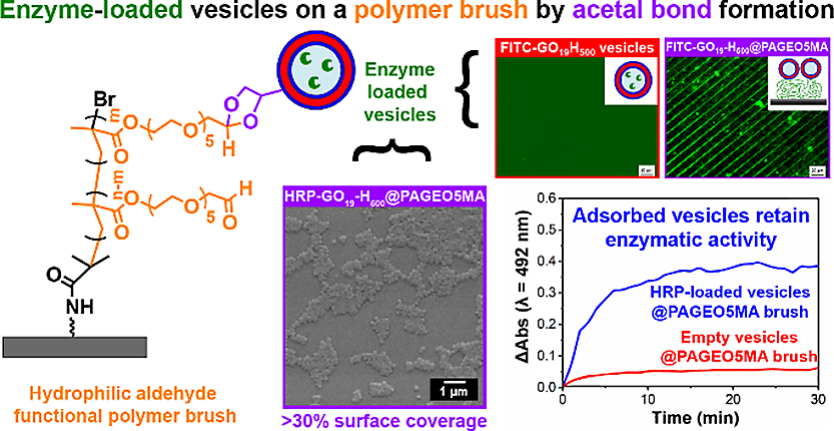

Compared to lipids, block copolymer vesicles are potentially robust nanocontainers for enzymes owing to their enhanced chemical stability, particularly in challenging environments. Herein we report that *cis*-diol-functional diblock copolymer vesicles can be chemically adsorbed onto a hydrophilic aldehyde-functional polymer brush via acetal bond formation under mild conditions (pH 5.5, 20 °C). Quartz crystal microbalance studies indicated an adsorbed amount, Γ, of 158 mg m^–2^ for vesicle adsorption onto such brushes, whereas negligible adsorption (Γ = 0.1 mg m^–2^) was observed for a control experiment conducted using a *cis*-diol-functionalized brush. Scanning electron microscopy and ellipsometry studies indicated a mean surface coverage of around 30% at the brush surface, which suggests reasonably efficient chemical adsorption. Importantly, such vesicles can be conveniently loaded with a model enzyme (horseradish peroxidase, HRP) using an aqueous polymerization-induced self-assembly formulation. Moreover, the immobilized vesicles remained permeable toward small molecules while retaining their enzyme payload. The enzymatic activity of such HRP-loaded vesicles was demonstrated using a well-established colorimetric assay. In principle, this efficient vesicle-on-brush strategy can be applied to a wide range of enzymes and functional proteins for the design of next-generation immobilized nanoreactors for enzyme-mediated catalysis.

## Introduction

Following seminal studies by Discher, Bates, and Eisenberg, block copolymer vesicles have become widely recognized as versatile synthetic delivery vehicles for potential biomedical applications.^1−6^ Their membranes are thicker and much more durable than those of lipid-based liposomes^2^: active vesicle payloads include anticancer drugs,^7^ DNA,^8^ RNA,^9^ magnetite nanoparticles,^10^ near-IR emitters,^11^ lectins,^12^ and enzymes.^13−16^ Autonomous motion of platinum-loaded vesicles has also been demonstrated using H_2_O_2_ as a fuel,^14^ while adsorption of vesicles onto a planar glass substrate has been achieved using Schiff base chemistry.^17^

In principle, diblock copolymer vesicles offer many potential applications in drug delivery, cell/organelle-mimicry, and for the design of enzymatic nanoreactors.^18−22^ Typically, such vesicles are prepared using traditional self-assembly techniques such as a solvent switch,^1^ temperature modulation,^23,24^ or thin film rehydration.^25^ However, these post-polymerization processing methods are normally restricted to dilute solution (<1% w/w) and hence are not suitable for industrial scale-up. Moreover, an organic cosolvent is often required for self-assembly, which may compromise the activity of payloads comprising delicate biological (macro)molecules.

Over the past decade or so, polymerization-induced self-assembly (PISA) has become widely recognized as a highly convenient route for the efficient preparation of various types of diblock copolymer vesicles.^26,27^ For aqueous PISA syntheses, this approach usually involves chain extension of a water-soluble precursor with a suitable vinyl monomer to produce a water-insoluble block.^28,29^ If the target degree of polymerization (DP) for the hydrophobic block is relatively high and the precursor block is sufficiently short, then well-defined vesicles can be obtained at a relatively high copolymer concentration (>20% w/w solids). During such PISA syntheses, a hydrophilic payload can be incorporated within the vesicles, which can be subsequently released on application of an appropriate stimulus.^15,16^ In principle, diblock copolymer vesicles prepared via aqueous PISA offer significant advantages for biomedical applications and catalysis. However, such polymerizations are typically conducted at around 70 °C using a thermal initiator, which can lead to denaturation of most proteins and enzymes.^30^ To avoid this problem, Armes and coworkers reported the in situ encapsulation of either silica nanoparticles or a model protein within diblock copolymer vesicles prepared via aqueous PISA.^15,31^ Such syntheses were conducted via reversible addition-fragmentation chain transfer (RAFT) aqueous dispersion polymerization at 40 °C using a low-temperature initiator, with essentially full monomer conversion being achieved within 8 h. Moreover, subsequent release of the encapsulated silica nanoparticles was achieved on cooling to 0–5 °C, which induced vesicle dissociation. Alternatively, Tan and coworkers reported the encapsulation of a model protein within vesicles via visible light-initiated RAFT aqueous dispersion polymerization conducted at 20 °C.^18,32,33^ Subsequently, O’Reilly, Gibson, and coworkers utilized the same approach to prepare protein- and enzyme-loaded nanoreactors.^13,14,34,35^

**Scheme 1:**
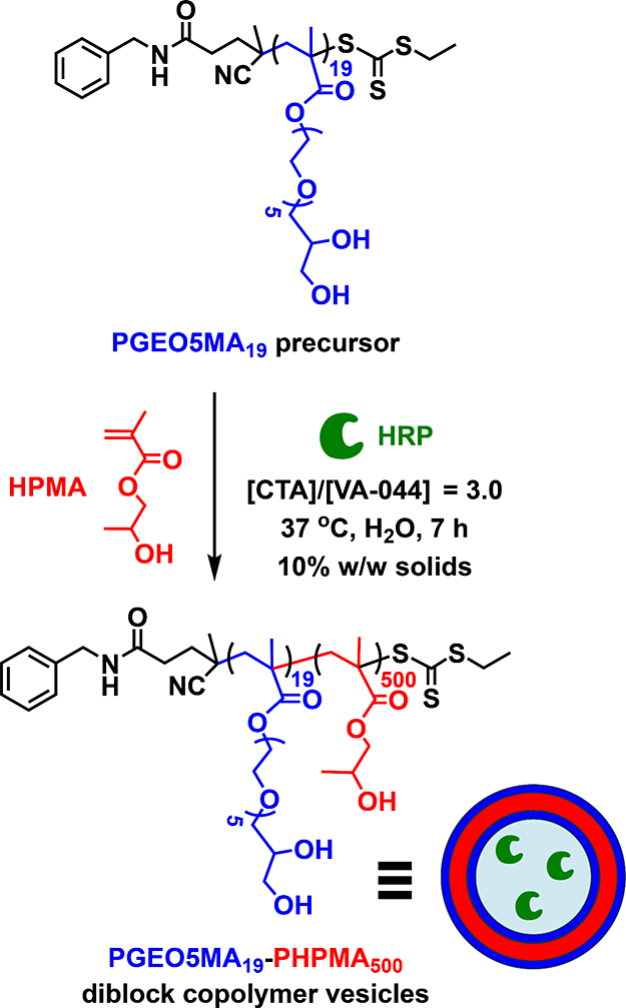
Synthetic Route for the Preparation of HRP-Loaded *cis*-Diol-Functional PGEO5MA_19_-PHPMA_500_ (GO_19_-H_500_) Vesicles via RAFT Aqueous Dispersion Polymerization of HPMA at 37 °C Targeting 10% w/w Solids An identical protocol was used to produce empty (enzyme-free) vesicles.

Herein we revisit a well-established aqueous PISA formulation^36^ to prepare *cis*-diol-functionalized diblock copolymer vesicles (see Scheme 1). Conducting such syntheses at 37 °C enables in situ encapsulation of a model enzyme (horse radish peroxidase, HRP). Recently, we reported the synthesis of an aldehyde-functionalized hydrophilic brush grafted from a planar silicon wafer.^37^ Such brushes can be decorated with *cis*-diol-functionalized vesicles via acetal bond formation in a mildly acidic aqueous solution. Adsorption of synthetic block copolymer nanoparticles (e.g., spheres or vesicles) onto polymer brushes have been reported by various mechanisms.^38−45^ However, to the best of our knowledge, this is the first example of vesicle adsorption via acetal chemistry, which is amenable to both mild reaction conditions and pH modulation. The extent of vesicle adsorption is monitored using scanning electron microscopy (SEM) and quartz crystal microbalance (QCM). Finally, a colorimetric assay is employed to assess whether the encapsulated enzyme remains catalytically active after chemical adsorption of the vesicles at the brush surface.

## Experimental

### Materials

All chemicals were used without further purification, unless otherwise stated. 4-Cyano-4-(ethylsulfanylthiocarbonyl)sulfanylpentanoic acid (CEPA) was prepared according to a literature protocol.^46^ 4-(Dimethylamino)pyridine (DMAP, 99%) was purchased from Alfa Aesar (UK). Ethyl acetate (≥99.0%), *N,N,N′,N″,N*″**-pentamethyldiethylenetriamine (PMDETA, >99.0%), hydrogen peroxide (35% v/v), and sodium hydroxide were purchased from Fisher Scientific, UK. Dichloromethane (DCM, 99.8%), *N*,*N*′**-dicyclohexylcarbodiimide (DCC, ≥99.0%), benzyl amine (≥99.5%), sodium periodate (NaIO_4_, ≥99.8%), (3-aminopropyl)triethoxysilane (APTES, >99%), triethylamine (TEA, 99%), 2-bromoisobutyryl bromide (BiBB, >99%), copper(II) chloride (CuCl_2_, 99.999%), ascorbic acid (AscAc, >98%), 4-4*′*-azobis(4-cyanovaleric acid (ACVA, ≥98.0%), 3,3′-dimethoxybenzidine (DMB, ≥98%), and d4-methanol (CD3OD, ≥99.8%) were purchased from Sigma-Aldrich Ltd. (UK). 2-Hydroxypropyl methacrylate (HPMA; mixture of 75 mol% 2-hydroxypropyl methacrylate and 25 mol% 2-hydroxyisopropyl methacrylate, >99%) was kindly donated by GEO Specialty Chemicals (Hythe, UK). GEO5MA monomer was prepared according to our recently reported experimental protocol.^47^ 2,2*′*-Azobis[2-(2-imidazolin-2-yl)propane]dihydrochloride (VA-044, 97%) was purchased from Wako Chemicals (Japan). Horseradish peroxidase (HRP) derived from Amoracia rusticana (type VI, essentially salt-free) was purchased from Sigma-Aldrich Ltd. (UK), divided into aliquots at 200 U mL^–1^ in deionized water, and stored at −20 °C prior to use. Deionized water (pH 6.8) was obtained using an Elga Elgastat Oprion 3A water purification system. Native oxide-coated silicon wafers were purchased from Pi-KEM, Tamworth, UK. Fluorescein 5(6)-isothiocyanate (>90%) and sodium phosphate dibasic salt (>99.5%) were purchased from Sigma-Aldrich and used as received.

### Synthetic Protocols

#### Synthesis of Bz-CEPA RAFT Agent

CEPA was reacted with benzyl amine using a DCC/DMAP catalyst.^48^ All glassware used for this amidation was dried overnight in a 200 °C oven. DMAP (32.5 mg, 0.27 mmol), DCC (0.98 g, 4.75 mmol), benzyl amine (0.15 g, 1.40 mmol), and CEPA (0.63 g, 2.38 mmol) were weighed into dry glass vials, which were sealed with rubber septa. A minimal amount (0.5–3.0 mL) of anhydrous DCM was used to dissolve each reagent. A two-necked 100 mL round-bottomed flask fitted with a condenser was sealed with a rubber septum and immersed in an ice bath. DMAP, benzyl amine, and CEPA were introduced into the flask in turn via syringe, and then, the DCC was slowly added dropwise with continuous stirring. The reaction mixture was allowed to warm up to 20 °C, while being maintained under a dry nitrogen atmosphere. Then, the reaction mixture was heated to reflux for 48 h and allowed to cool before removing the insoluble *N*,*N*′**-dicyclohexylurea byproduct by filtration. Solvent was removed under vacuum, and the crude product was purified by column chromatography using a silica stationary phase and a mobile phase comprising 60:40 ethyl acetate/hexane for the first fraction, followed by ethyl acetate for the second fraction. The mean degree of amidation was calculated to be 103 ± 3% by end-group analysis using ^1^H NMR spectroscopy (the integrated CEPA signals at 3.40, 2.52, 1.92, and 1.39 ppm were compared to the five aromatic benzyl protons at 7.23–7.47 ppm).

#### Synthesis of PGEO5MA_19_ Precursor via RAFT Solution Polymerization of GEO5MA

The RAFT solution polymerization of GEO5MA was conducted according to a literature protocol.^36^ A solution of Bz-CEPA (0.200 g, 0.57 mmol, 1.0 eq) in anhydrous ethanol (10.0 g, 50% w/w solids) was added to a round bottom flask containing GEO5MA monomer (4.35 g, 11.4 mmol, 20 eq) and ACVA (32.0 mg, 0.11 mmol, 0.20 eq). This reaction mixture was purged with dry nitrogen gas for 20 min, and the flask was then immersed in an oil bath set at 70 °C for 3 h. To quench the polymerization, the flask was removed from the oil bath and its contents were exposed to air while cooling to 20 °C. The crude precursor was precipitated into excess diethyl ether, isolated by filtration, and redissolved in methanol. This purification step was repeated, and the resulting homopolymer was dried overnight in a vacuum oven at 25 °C to produce a yellow viscous liquid. End-group analysis by ^1^H NMR spectroscopy (400 MHz, CD3OD) indicated a mean DP of 19 for this precursor, which is denoted hereafter as either PGEO5MA_19_ or GO_19_.

#### Synthesis of PGEO5MA_19_-PHPMA_500_ (GO_19_-H_500_) Diblock Copolymer Vesicles via RAFT Aqueous Dispersion Polymerization

The synthesis of GO_19_-H_500_ vesicles via RAFT aqueous dispersion polymerization of HPMA was conducted using the above GO_19_ precursor.^13,40^ More specifically, HPMA (0.180 g, 1.25 mmol, 500 eq), GO_19_ precursor (19.0 mg, 2.51 μmol, 1.0 eq), VA-044 initiator (0.27 mg, 0.83 μmol, 0.33 eq; [GO_19_]/[VA-044] molar ratio = 3.0), and deionized water (1.79 mL, 10% w/w solids) were placed in a glass vial equipped with a magnetic stirrer bar. Once this aqueous solution was purged with a stream of N_2_ gas for 20 min, the vial was immersed in an oil bath set at 37 °C. After 7 h, the polymerization was quenched by removing the vial from the oil bath and cooling to 20 °C while exposing the reaction solution to air. The final HPMA conversion exceeded 99%, as judged by ^1^H NMR spectroscopy (the integrated vinyl signals at 3.4–4.2 ppm were compared to the integrated methacrylic backbone proton signals at 0.97–2.19 ppm).

#### Synthesis of HRP-Loaded PGEO5MA_19_-PHPMA_500_ (GO_19_-H_500_) Diblock Copolymer Vesicles via RAFT Aqueous Dispersion Polymerization

The synthesis of GO_19_-H_500_ vesicles via RAFT aqueous dispersion polymerization of HPMA was conducted using the above protocol in the presence of HRP.^13,40^ HPMA (0.180 g, 1.25 mmol, 500 eq), GO_19_ precursor (19.0 mg, 2.51 μmol, 1.0 eq), VA-044 initiator (0.27 mg, 0.83 μmol, 0.33 eq; [GO_19_]/[VA-044] molar ratio = 3.0), 200 μL of a 200 U mL^–1^ aqueous solution of HRP, and deionized water (1.59 mL, 10% w/w solids, pH 6) were placed in a glass vial equipped with a magnetic stirrer bar. After this aqueous solution was purged with dry N_2_ for 20 min, the vial was immersed in an oil bath set at 37 °C. After 7 h, the polymerization was quenched by removing the vial from the oil bath and cooling to 20 °C while exposing the reaction solution to air. The milky white dispersion was diluted to 1.0% w/w using 100 mM phosphate buffer solution solution (PB, pH 5.5), and non-encapsulated enzyme was removed by three successive centrifugation-redispersion cycles in which each supernatant was replaced with fresh PB solution. The final HPMA conversion exceeded 99%, as judged by ^1^H NMR spectroscopy (the integrated vinyl signals at 3.4–4.2 ppm were compared to the integrated methacrylic backbone protons at 0.97–2.19 ppm).

#### Labeling of Empty GO_19_-H_500_ Diblock Copolymer Vesicles with Fluorescein Isothiocyanate (FITC)

Empty GO_19_-H_500_ vesicles were labeled with fluorescein isothiocyanate (FITC). A 1.0% w/w aqueous dispersion of GO_19_-H_500_ vesicles [30.0 mg (0.38 μmol) copolymer in 3.0 mL PB at pH 5.5] and FITC [0.30 mg (0.75 μmol), i.e., [HPMA]/[FITC] molar ratio = 250] was stirred in a glass vial at 23 °C for 72 h. The FITC-labeled vesicles were subsequently purified by two centrifugation/resuspension cycles to remove any unreacted dye, with each supernatant being replaced with fresh PB solution (pH 5.5) prior to further analysis.

#### Surface Functionalization of Substrates with ATRP Initiator Groups

ATRP initiator-functionalized silicon wafers (or glass coverslips) were prepared using a modified literature protocol.^37,40^ Briefly, ∼1 × 1 cm pieces of wafer were subjected to UV-ozone cleaning for 30 min using a Bioforce Nanosciences ProCleaner (BioForce Nanosciences, USA). The resulting wafers were immersed in 0.5 M NaOH and rinsed with deionized water and ethanol. The chemical vapor deposition of APTES onto clean wafers was conducted for 30 min at 22 °C. Then, the APTES-coated wafers were removed and dried in a 110 °C oven for 30 min, prior to immersion in a solution of 2-bromoisobutyryl bromide (BiBB) in dichloromethane in the presence of triethylamine (conditions: DCM/TEA/BiBB = 400:1:1 by volume) and the amidation reaction was allowed to proceed for 1 h at 22 °C. Finally, the resulting ATRP initiator-functionalized substrates were rinsed with (i) ethanol and (ii) deionized water before drying using a stream of compressed air.

#### Preparation of Patterned Initiator Surfaces for the Synthesis of Patterned Brushes for Brush Growth Kinetics

A Coherent Innova 300C FreD frequency-doubled argon ion laser (Coherent UK, Ely, UK) with an emission wavelength of 244 nm was used for the UV photodegradation experiments. The energy density was measured prior to each experiment, and the dose was calculated accordingly. Micropatterned brushes were obtained by irradiating uniform BiBB layers on either silicon or glass wafers using a copper mesh electron microscopy grid (Agar, Cambridge, UK) as a photomask. 2-Bromoisobutyryl initiator groups were photocleaved by irradiation to a total dose of 35 J cm^–2^. This protocol is a modified version of previously reported protocols.^49^

#### Preparation of Patterned Initiator Surfaces for the Synthesis of Patterned Brushes for Adsorption Studies

A UV lamp (Analytik Jena US, UVP 3UV, 8 W) with an emission wavelength of 254 nm was used to pattern ATRP initiator-functionalized silicon wafers for vesicle adsorption studies. Micropatterned surfaces were obtained with a similar copper mesh grid mask as above. Substrates were exposed to UV irradiation for 32 h.

#### Synthesis and Functionalization of PGEO5MA Brushes and Oxidation to PAGEO5MA

PGEO5MA brushes were grown from either silicon wafers or glass coverslips using surface-initiated ARGET ATRP (SI-ARGET ATRP).^37,40^ In a typical experiment, the desired initiator-functionalized substrate was placed in an aqueous solution comprising GEO5MA (6.48 g, 1.4 M), copper(II) chloride (2.3 mg, 1.4 mM), PMDETA (14.8 mg, 7.1 mM), and deionized water (6.60 g) to afford a final GEO5MA concentration of 45% v/v. Ascorbic acid (30 mg, 14.2 mM) was then added to this aqueous solution, which was stirred for 10 min at 22 °C. The ensuing surface-initiated polymerization was allowed to proceed for 2 h at 22 °C. Each wafer was then removed from the reaction solution, rinsed thoroughly with ethanol and deionized water, and dried using a stream of compressed air.

#### Selective Oxidation of a PGEO5MA Brush to Produce a PAGEO5MA Brush

Selective oxidation of a *cis*-diol-functional PGEO5MA brush to obtain an aldehyde-functionalized PAGEO5MA brush was conducted using an optimized literature protocol.^37,40^ PGEO5MA brush-decorated wafers (or glass coverslips) were immersed in an aqueous solution of NaIO_4_ (3.0 g dm^–3^) for 30 min at 22 °C. Then, these planar substrates were removed and rinsed thoroughly with ethanol and deionized water, followed by drying using a stream of compressed air.

#### Adsorption of GO_19_-H_500_, FITC-GO_19_-H_500_, or HRP-Loaded GO_19_-H_500_ Vesicles onto a PAGEO5MA Brush

A PAGEO5MA-functionalized silicon wafer (or glass coverslip) was immersed into a 1.0% w/w aqueous dispersion containing either empty GO_19_-H_500_ vesicles or HRP-loaded GO_19_-H_500_ vesicles in 100 mM PB solution at pH 5.5, and vesicle adsorption was allowed to proceed overnight for a minimum of 16 h at 22 °C. Then, the planar substrate was removed from the aqueous dispersion, rinsed with 100 mM PB solution (pH 5.5), and dried using a stream of compressed air.

### Characterization Techniques

#### Nuclear Magnetic Resonance Spectroscopy (NMR)

^1^H NMR spectra were recorded at 400 MHz using a Bruker Ascend 400 spectrometer. Chemical proton shifts are reported as δ in parts per million (ppm) and are expressed relative the residual solvent peak at 3.31 ppm when using CD_3_OD or 5.29 ppm when using CD_2_Cl_2_.

#### Size Exclusion Chromatography (SEC)

Polymer molecular weights and dispersities were determined using an Agilent 1260 Infinity GPC system equipped with an Agilent guard column and two Agilent PLgel 5 μm Mixed-C columns connected in series, a differential refractive index (RI) detector, and a UV–visible detector set to 305 nm, which is the wavelength for maximum absorption by the trithiocarbonate-based RAFT agent. The SEC eluent was HPLC grade DMF containing 10 mM LiBr at 60 °C at a flow rate of 1.0 mL min^–1^. The number-average molecular weight (*M*_n_), weight-average molecular weight (*M*_w_), and dispersity (*Đ* = *M*_w_/*M*_n_) were calculated using a series of near-monodisperse poly(methyl methacrylate) (PMMA) calibration standards.

#### Dynamic Light Scattering (DLS)

Particle size distributions were determined using a Malvern Zetasizer Nano ZS instrument equipped with a 4 mW He–Ne 633 nm laser and an avalanche photodiode detector. Dilute aqueous vesicle dispersions (0.1% w/w) were analyzed at 22 °C via back-scattered light detection at 173°. Malvern Zetasizer software was used to calculate the hydrodynamic *z*-average diameter (*D*_h_) via the Stokes–Einstein equation, which assumes perfectly monodisperse, non-interacting spherical particles. Data were averaged over at least three consecutive runs with at least ten measurements being recorded for each run.

#### Transmission Electron Microscopy (TEM)

Conventional TEM images were recorded at an acceleration voltage of 100 kV using a Philips CM100 microscope equipped with a Gatan 1k CCD camera. As-synthesized aqueous vesicle dispersions were diluted at 22 °C to generate 0.1% w/w dispersions, which were then deposited onto freshly glow-discharged carbon-coated copper/palladium grids via micropipette at 25 °C for approximately 60 s. Finally, 8 μL of a 0.75% w/v aqueous solution of uranyl formate was deposited onto each sample-loaded grid for 60 s and then carefully blotted to remove excess stain prior to drying under vacuum.

#### Cryogenic Transmission Electron Microscopy (Cryo-TEM)

Cryo-TEM images were recorded using an FEI Tecnai Arctica microscope operating at an acceleration voltage of 200 kV. Samples were prepared by depositing 5 μL of a 0.5% w/w aqueous dispersion comprising either empty or purified HRP-loaded vesicles onto a plasma-treated Quantifoil carbon-coated holey copper grid, followed by blotting for approximately 4 s and then plunging into a pool of liquid ethane to rapidly vitrify the sample using a Leica EM GP automatic plunge freezer (25 °C, 99% humidity). Transfer of the vitrified grids into a precooled cryo-TEM holder was performed at −196 °C prior to analysis.

#### Scanning Electron Microscopy (SEM)

SEM images were acquired using an FEI Inspect F field emission scanning electron microscope operating at an acceleration voltage of 5–15 kV. Samples comprising either empty or HRP-loaded vesicles were prepared by drying the desired 0.1% w/w aqueous dispersion onto a silicon wafer at 25 °C. Brush-coated silicon wafers were imaged directly after their purification. The sample-loaded silicon wafers were mounted on aluminum stubs using adhesive carbon tabs. Silver paint was applied to two edges of the mounted silicon wafers prior to sputter coating with a thin gold overlayer to prevent charge build-up. Surface coverages were estimated for vesicle-decorated brushes using ImageJ software by averaging across multiple images recorded for each sample.

#### Atomic Force Microscopy (AFM)

AFM imaging was performed using a Bruker Nanoscope VIII Multimode Atomic Force Microscope equipped with a ‘J’ scanner. Silicon cantilevers (OTESTPA-R3, Bruker, UK) with a nominal spring constant of 26 N·m^–1^ and a tip radius of 9 nm were used for tapping mode imaging. AFM images were recorded at 22 °C for dried PGEO5MA and PAGEO5MA brushes (both patterned and unpatterned) plus a PAGEO5MA brush decorated with HRP-loaded GO_19_-H_500_ vesicles.

#### Fluorescence Microscopy

Fluorescence microscopy images were recorded for (i) a 1.0% w/w aqueous dispersion of FITC-labeled GO_19_-H_500_ vesicles and (ii) vesicles adsorbed onto an aldehyde-functionalized brush grown from a glass coverslip using a Zeiss Axio Scope A1 microscope equipped with a Zeiss Axio ICm1 camera. Fluorescence images were obtained using an LED radiation source combined with filter set 38 (excitation λ = 470 nm, emission λ > 525 nm).

#### Ellipsometry Studies of Dry and Solvated Polymer Brushes

Dry and solvated thicknesses for each brush and selected vesicles-on-brush systems were measured via spectroscopic ellipsometry using a J. A. Woollam M2000 V ellipsometer at a fixed angle of incidence of 75° normal to the sample surface. A wavelength range of 370–1000 nm was used to obtain two ellipsometric parameters (Ψ and Δ). Measurements were only performed for brushes grown from planar silicon wafers. Dry measurements were performed in air at 20 °C. Solvated brush measurements were made using a large volume, open-top liquid cell with optical glass windows fixed at 75°. Each substrate was positioned within the cell before filling with water at 20 °C. The ellipsometer setup provided a relatively large sampling area of approximately 0.5 cm × 1 cm, which corresponds to around 75% of the total area of each sample. Thus, such measurements expected to be representative of the whole sample.

Data analysis and modeling were performed using Woollam CompleteEase software, which fits the Ψ and Δ values. Each system (i.e., dry brush, solvated brush, solvated vesicles-on-brush, or dried vesicles-on-brush) required a unique model to obtain satisfactory data fits. For dry brush measurements, the interfacial structure consisted of polymer and silica slabs, each describing their respective refractive index and thickness, plus a silicon backing layer. The polymer layer was treated as a Cauchy layer with the following parameters: *A*_n_ = 1.4615, *B*_n_ = 0.00514 μm^–2^, and *C*_n_ = 0 μm^–4^. The silica layer thickness was allowed to vary between 1 and 3 nm, with a typical value being ∼1.5 nm. Solvated brushes were analyzed using a similar model. However, in this case, the fronting medium was water, whereas air was used for the dry measurements. The hydrated brush layer was modeled using an effective medium approximation (EMA) where optical properties are calculated as a weighted average according to the relative volume fractions of the polymer plus water (assuming a Cauchy layer with the above parameters). The thickness and polymer volume fraction were allowed to vary during data fitting.

**Figure 1:**
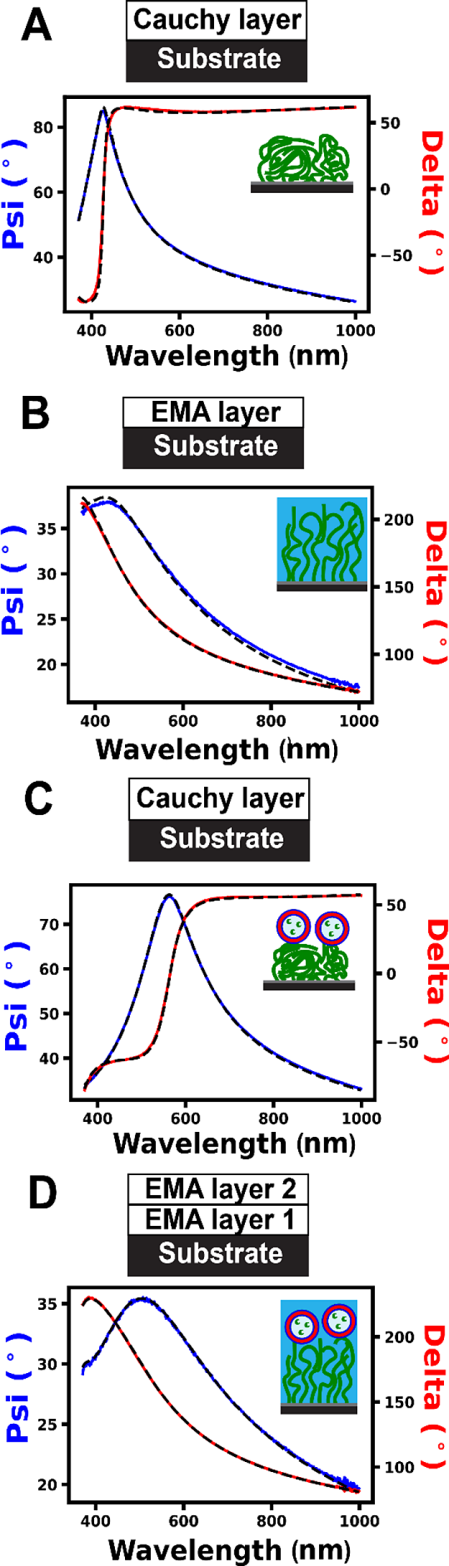
Representative ellipsometry data (solid lines) and fits (dashed) with schematic cartoons for the four interfacial structures and their corresponding ellipsometry models. Psi values are shown in blue, while delta values are shown in red. (A) Dry PAGEO5MA brush represented in the ellipsometry model by a single Cauchy layer with variable thickness (Cauchy parameters in the main text). (B) Hydrated PAGEO5MA brush represented by a single effective medium approximation (EMA) layer with a variable thickness and water content. (C) Dry vesicle-on-brush represented in the ellipsometry model by a single Cauchy layer with variable thickness (same Cauchy parameters as used for panel A). (D) Solvated vesicle-on-brush system, which required two EMA layers to adequately fit the data. Each layer comprised a mixture of polymer and water and the thickness and composition was allowed to vary in both layers.

For the dry vesicles-on-brush systems, a single Cauchy layer produced a satisfactory data fit. However, for the hydrated vesicles-on-brush systems, a more complex model was required. Two almost identical EMAs comprising water and polymer were required. These two EMAs varied in terms of their composition (i.e., water volume fraction and solvated brush thickness). A schematic cartoon of each interfacial model and a typical data fit is shown in Figure 1.

#### QCM Measurements

Quartz crystal microbalance sensors coated with a 50 nm silica overlayer (QSX 303, ∼5 MHz fundamental frequency) were purchased from Q-Sense (Sweden). Each sensor was cleaned according to the manufacturer’s instructions. This protocol involved (i) UV/O_3_ treatment for 15 min (Bioforce UV/O_3_ cleaner, ∼9 mW cm^–2^, λ = 254 nm), (ii) exposure to 2% w/w sodium dodecylsulfate solution for 30 min, (iii) copious rinsing with deionized water and drying under N_2_, and (iv) a final UV/O_3_ treatment for 15 min. The resulting substrates were functionalized with initiator prior to surface-initiated polymerization to produce brush-coated substrates using the protocols given below.

QCM measurements were performed using an openQCM NEXT instrument (Novatech S.r.l., Italy) equipped with a temperature-controlled cell connected to a Masterflex Digital Miniflex peristaltic pump (Cole-Parmer Instrument Company, UK). A rapid flow of ethanol (2.0 mL min^–1^) was used to wet the sensor surface.

Once all bubbles had been removed, water and then PB solution were flown through the cell until the sensor frequency exhibited a drift of less than 1 Hz min^–1^. This typically occurred within 1 h. Once a stable signal was obtained, a 1% w/w aqueous dispersion of vesicles was passed through the cell at a flow rate of 0.1 mL min^–1^ (minimum flow volume = 3 mL). Once any signal drift had abated, PB solution was passed through the cell at the same flow rate. All QCM experiments were performed at 25 °C. The adsorbed amount can be calculated using the Sauerbrey equation, which relates the change in resonant frequency, Δ*f* (Hz), to the change in adsorbed mass per unit area, *m*.
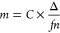
where *C* is a sensitivity constant −0.177 mg m^–2^ Hz^–1^ and *n* is the overtone number. The third harmonic (*n* = 3) was used to calculate the adsorbed amount to avoid experimental artifacts associated with the fundamental harmonic.

#### Enzymatic Activity Assay for Either Empty or HRP-Loaded GO_19_-H_500_ Vesicles

After purification via dialysis to remove nonencapsulated enzyme, either empty or HRP-loaded GO_19_-H_500_ vesicles were diluted to 0.5% w/w using 100 mM PB solution (pH 5.5) and 120 μL aliquots of this dilute vesicle dispersion were added to individual wells of a 96-well plate. Then 20 μL of 100 mM PB solution (pH 5.5) and 40 μL of a 2 mM aqueous solution of DMB were added to each well, followed by addition of 20 μL of a 35% w/w aqueous hydrogen peroxide solution. The resulting change in absorbance at 492 nm was monitored over 30 min using a microplate reader (Accuris Instruments SmartReader MR9600).^13,35^ The enzyme activity for both free and encapsulated HRP was determined by monitoring the oxidation of DMB at 22 °C. In the presence of H_2_O_2_, HRP produces a highly reactive iron-oxygen complex. This species oxidizes DMB, generating radicals which then couple to form a red dimer product.^50^ This colorimetric reaction enables the enzyme activity to be assessed for each sample.

A similar protocol was adapted to evaluate the activity of the free enzyme. Various concentrations of HRP in 100 mM PB solution 5.5 (120 μL) were mixed with 20 μL of 100 mM PB solution 5.5 using a 96-well plate. Subsequently, 40 μL of a 2 mM aqueous solution of DMB was introduced, followed by addition of 20 μL of a 35% w/v aqueous solution of H_2_O_2_. All measurements were conducted four times, and average values are presented.

Enzymatic activity assay for GO_19_-H_500_ vesicles adsorbed onto a PAGEO5MA brush: A spectrophotometric microplate reader (Accuris Instruments SmartReader MR9600) was used to evaluate the enzymatic activity of either empty or HRP-loaded vesicles immobilized on PAGEO5MA-coated glass coverslips. After washing the vesicle-functionalized coverslips with 100 mM PB solution to remove any non-adsorbed vesicles, each coverslip was placed parallel to the light beam in a 96-well plate and immersed in 105 μL of 100 mM PB solution (pH 5.5) and 30 μL of a 2 mM aqueous solution of DMB, followed by 15 μL of a 35% w/v aqueous solution of H_2_O_2_. The resulting change in absorbance at 492 nm for the aqueous solution within the well was monitored for 30 min using the microplate reader.^13,35^

## Results and Discussion

### Synthesis of the RAFT Agent and Water-Soluble Non-ionic Precursor

To prevent in situ denaturation, mild reaction conditions are essential for the encapsulation of enzymes within diblock copolymer vesicles during aqueous PISA. First, a benzyl-capped trithiocarbonate-based RAFT agent was prepared for the synthesis of a suitable non-ionic water-soluble precursor. This is important to ensure that the target vesicles do not exhibit any unwanted pH-responsive behavior owing to end-group ionization.^51^ Accordingly, benzyl amine was reacted with 4-cyano-4-(((ethylthio)carbonothioyl)thio)pentanoic acid (CEPA) via DCC/DMAP-catalyzed amidation. Figure S1 shows the ^1^H NMR spectra recorded for the CEPA RAFT agent, benzyl amine, and the final Bz-CEPA RAFT agent used in this study. Essentially, full amidation was confirmed by comparing the integrated CEPA-related signals at 3.3–3.42 ppm (CH_3_CH_2_S), 2.42–2.60 ppm (C(CH_3_)(C≡N)CH_2_CH_2_(C=O)), 1.92 ppm (see methyl signal *c* in Figure S1), and 1.33–1.41 ppm (see methyl signal *a* in Figure S1) to the five aromatic protons at 7.27–7.43 ppm assigned to the benzyl end-group.

**Figure 2:**
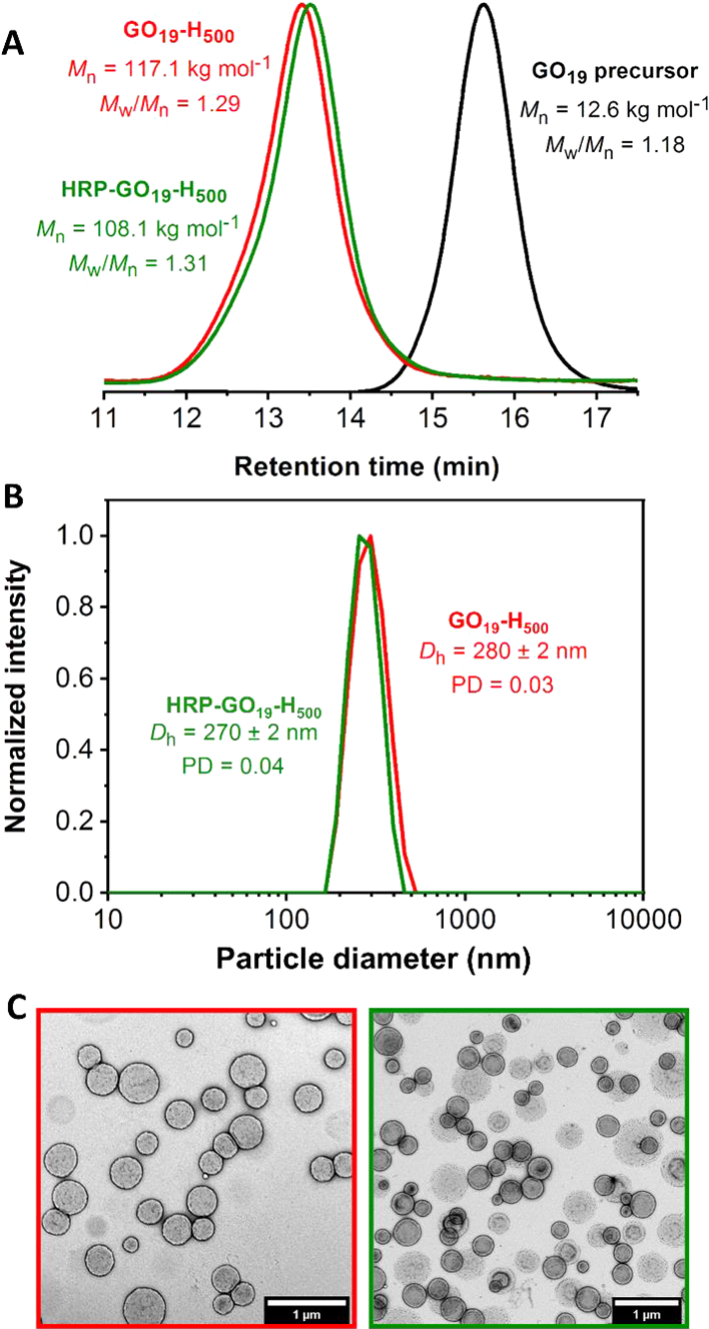
(A) Normalized SEC data recorded for the GO_19_ precursor (black curve), GO_19_-H_500_ diblock copolymer (red curve), and the GO_19_-H_500_ diblock copolymer prepared in the presence of HRP (green curve). (B) DLS data obtained for empty (red curve) and HRP-loaded (green curve) GO_19_-H_500_ vesicles. (C) Representative TEM images (uranyl formate stain) recorded for GO_19_-H_500_ vesicles (red border image—left) and HRP-loaded GO_19_-H_500_ vesicles (green border image—right).

Following a modified literature protocol,^36^ a *cis*-diol-functional PGEO5MA_19_ (GO_19_) precursor was prepared via RAFT solution polymerization of GEO5MA in ethanol at 70 °C using the Bz-CEPA RAFT agent (Scheme 1). Compared to our prior study,^36^ a relatively low DP was targeted for the PGEO5MA precursor because this is known to favor vesicle formation.^52^ A final GEO5MA conversion of 95% (corresponding to a mean DP of 19) was achieved within 3 h, as determined by ^1^H NMR analysis in CD3OD (Figure S2). SEC analysis of the GO_19_ precursor indicated an apparent number-average molecular weight (*M*_n_) of 12.6 kg mol^–1^ and a relatively low dispersity (*M*_w_/*M*_n_ = 1.18) (see Figure 2A). This suggests a well-controlled RAFT polymerization.

### Synthesis of Diblock Copolymer Vesicles and Enzyme Encapsulation

The water-soluble non-ionic GO_19_ precursor was chain-extended via RAFT aqueous dispersion polymerization of HPMA at 37 °C using a low-temperature azo initiator at pH 6 to obtain GO_19_-H_500_ diblock copolymer vesicles at 10% w/w solids. As expected, the rate of HPMA polymerization was relatively slow under these mild conditions.^15^ Nevertheless, more than 99% was achieved within 7 h, as judged by the substantial attenuation in the vinyl monomer proton signals at δ 6.2–6.6 ppm in an ^1^H NMR spectrum recorded for the final reaction mixture (Figure S2). Size exclusion chromatography (SEC) studies indicated a high chain extension efficiency (i.e., minimal GO_19_ precursor contamination) and a relatively low dispersity (*M*_w_/*M*_n_ = 1.29) for the diblock copolymer chains (Figure 2A), while dynamic light scattering (DLS) analysis revealed a narrow particle size distribution with a hydrodynamic diameter of 280 nm and a DLS polydispersity (PDI) of 0.03 (Figure 2B). Furthermore, transmission electron microscopy (TEM) studies confirmed the formation of a pure vesicular morphology (Figure 2C).

Since the HPMA polymerization is conducted under mild conditions, it should be feasible to encapsulate relatively delicate payloads such as enzymes into vesicles during PISA without inducing any denaturation. In the present study, this approach is exemplified using HRP. Importantly, the presence of this enzyme had no discernible impact on the polymerization, with full HPMA conversion, a high chain extension efficiency, and a similarly low dispersity being observed (Figures 2A and S2). The resulting HRP-loaded vesicles were purified via multiple centrifugation/redispersion cycles (13,000 rpm for 10 min per cycle) to remove non-encapsulated enzyme. Each successive aqueous supernatant was replaced with a 100 mM PB solution at pH 5.5. DLS studies confirmed that the particle size distribution obtained for the HRP-loaded GO_19_-H_500_ vesicles was comparable to that obtained for the corresponding empty GO_19_-H_500_ vesicles (Figure 2B), while TEM studies indicated a well-defined vesicular morphology in the presence of the enzyme (Figure 2C). In addition, cryo-TEM studies were undertaken to examine the vesicles in their hydrated form in the absence of any heavy metal stain (Figure S3). Cryo-TEM images were in good agreement with conventional TEM images and digital image analysis of the former indicated mean membrane thicknesses of 30 ± 1 and 31 ± 1 nm for the empty and HRP-loaded vesicles, respectively. In summary, the presence of the enzyme has no discernible effect on the formation of GO_19_-H_500_ vesicles via aqueous PISA under the stated reaction conditions.

### Synthesis of Vesicles-On-Brush Systems

**Scheme 2:**
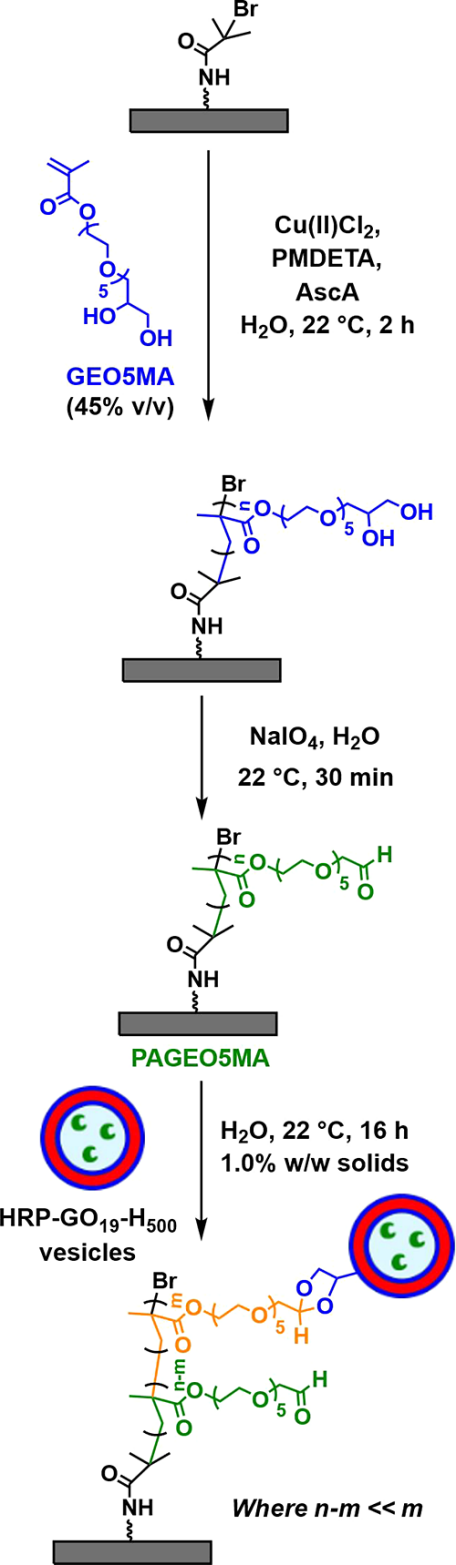
(i) Preparation of a *cis*-Diol-Functional PGEO5MA Precursor Brush via SI-ARGET ATRP Using Either a Planar Silicon Wafer or a Glass Slide, (ii) Selective Oxidation to Produce the Corresponding Aldehyde-Functional PAGEO5MA Brush, and (iii) Its Subsequent Exposure to Either GO_19_-H_500_ or HRP-GO_19_-H_500_ Vesicles to Produce Vesicle-On-Brush Systems

Having demonstrated the successful encapsulation of HRP within PGEO5MA_19_-PHPMA_500_ vesicles, we then examined their chemical adsorption at a model soft interface. More specifically, we utilized the *cis*-diol functionality of the PGEO5MA steric stabilizer chains expressed at the outer surface of the vesicles to form acetal bonds with an aldehyde-functionalized hydrophilic polymer brush (Scheme 2).^37^ Such chemistry is well-established and is known to proceed under mild conditions in aqueous media.^39,53^ In view of the enzyme-loaded vesicles described herein, this new vesicles-on-brush system is expected to offer potential biotechnology applications in the context of microfluidics and flow chemistry.^38,54^ Accordingly, a hydrophilic PGEO5MA brush was first grown from a 2-bromoisobutyryl bromide (BiBB)-functionalized silicon wafer (or glass coverslip) via SI-ARGET-ATRP. This surface polymerization of GEO5MA was conducted under ambient conditions without any degassing step to remove dissolved oxygen. A mean dry brush thickness of approximately 120 nm was determined by ellipsometry. Subsequently, selective oxidation of this precursor using NaIO_4_ produced an aldehyde-functionalized PAGEO5MA brush with a relatively high degree of functionalization.^37,47^ This model reactive brush was then exposed to either empty or HRP-loaded vesicles to produce the corresponding vesicles-on-brush systems, as shown in Scheme 2.

**Figure 3:**
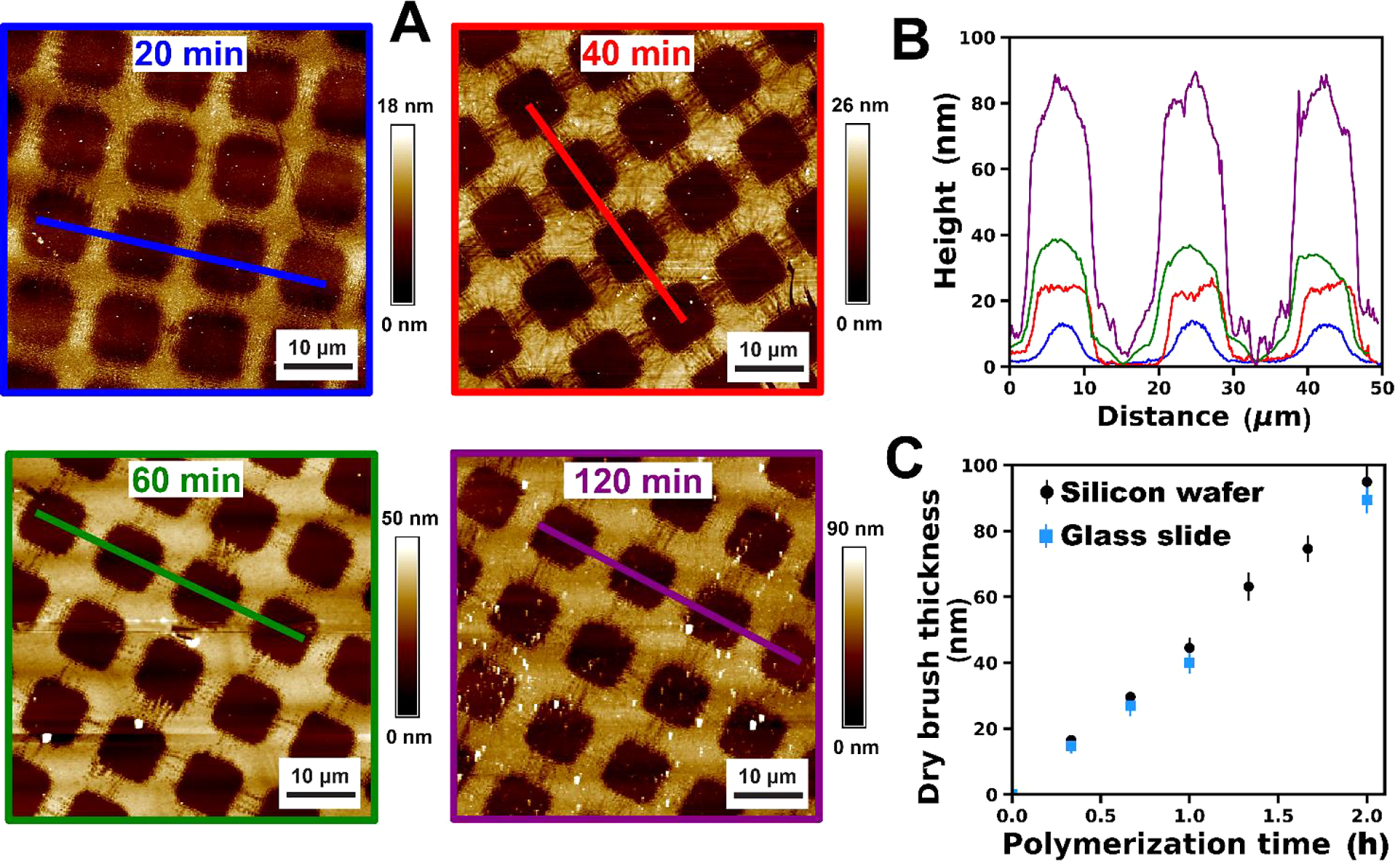
(A) Representative AFM images recorded for PGEO5MA brushes grown from patterned initiator films on planar glass slides for various polymerization times. (B) Line sections through AFM images of patterned brushes. (C) Increase in the dry brush thickness with polymerization time for PGEO5MA brushes grown from a planar silicon wafer and a glass slide. Dry brush thicknesses were determined by ellipsometry analysis for planar silicon wafers and from AFM images for glass slides (ellipsometry thickness data reproduced from Brotherton et al.^37^).

We briefly compared the kinetics of PGEO5MA brush growth from a glass slide and a planar silicon wafer, respectively. Unfortunately, modeling ellipsometry data from glass substrates can be problematic owing to the low reflectivity of the glass substrate plus backside reflections. Instead, dry brush thicknesses of PGEO5MA brushes grown from glass slides were determined from atomic force microscopy (AFM) height images recorded for surface-patterned brushes. Patterned initiator layers were prepared by UV-induced cleavage of the C–Br bond using a TEM grid as a mask. Surface-initiated polymerization was then performed using the resulting patterned initiator layers to produce the corresponding patterned brushes (see Figure 3A). A representative step height profile is provided in Figure 3B. As expected, the mean brush thickness increases linearly with polymerization time (Figure 3C). Importantly, PGEO5MA brushes grown from silicon wafers and glass slides exhibit very similar dry brush thicknesses for reaction times of at least 2 h (Figure 3C). Hence essentially the same mean brush thickness is produced within a given reaction time when using either substrate. Ellipsometry was also used to monitor the degree of swelling of PGEO5MA brushes immersed in water. Hydration of a dry brush (initial thickness = 120 nm) produced a final hydrated brush thickness of approximately 190 nm (Table S1). This corresponds to a swelling ratio of ∼1.6, which is comparable to that reported for other well-known hydrophilic polymer brushes.^55−58^ An excellent fit to the corresponding ellipsometric data is shown in Figure 1. It is well-known that the solvated brush/dry brush swelling ratio in a good solvent is proportional to the brush grafting density.^59,60^ We have used an approach reported by Jordan et al.^61^ to estimate a mean DP of 2600 and a brush grafting density of 0.5 chains nm^–2^, which is comparable to that obtained for other brush systems.^62,63^ This analysis assumes a monotonic, parabolic brush profile and a Kuhn length of 7 Å.

Selective NaIO_4_ oxidation of this hydrated PGEO5MA brush for 30 min at 22 °C produced an aldehyde-functionalized PAGEO5MA brush.^37,40,47^ The dry brush thickness was determined for each sample following NaIO_4_ treatment. A mean reduction in brush thickness of ∼ 8% was observed, which corresponds to the mass loss expected for full oxidation of PGEO5MA to PAEGO5MA (Figure S1).^37,40,64,65^ SEM studies indicated a smooth featureless surface morphology with no significant topographical features other than those introduced by gold sputter coating, which is essential to minimize sample charging.

We have recently reported the chemical adsorption of aldehyde-functionalized spherical nanoparticles onto an amine-functionalized planar polymer brush.^40^ This was achieved via imine bond formation, which is an example of dynamic covalent chemistry.^66−69^ Initially, we explored this approach for the design of vesicles-on-brush systems. Unfortunately, the NaIO_4_ reagent required to introduce aldehyde functionality proved to be incompatible with the HRP-loaded vesicles, which suffered oxidative degradation and partial loss of their morphology. Hence an alternative strategy was adopted based on acetal bond formation (Scheme 2). In this case, NaIO_4_ is solely used to oxidize the brush chains, so chemical degradation of the HRP-loaded vesicles is avoided.

**Figure 4:**
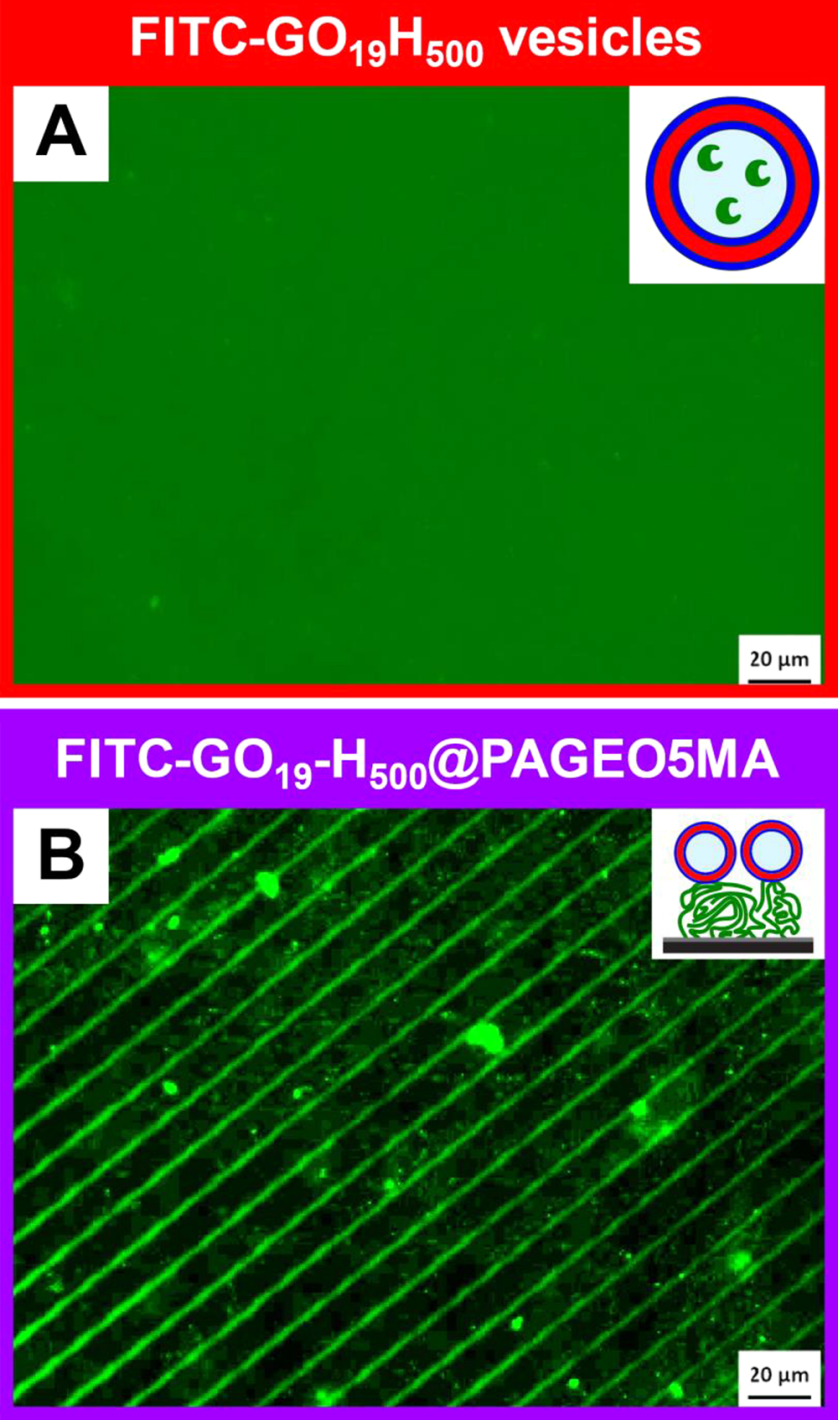
Fluorescence microscopy images recorded for (A) a 1.0% w/w aqueous dispersion of FITC-labeled GO_19_H_500_ vesicles in solution and (B) the vesicle-on-brush system: FITC-labeled GO_19_H_500_ vesicles adsorbed at pH 5.5 on a 116 nm PAGEO5MA brush grown from a patterned initiator film on a planar silicon wafer.

First, empty GO_19_-H_500_ vesicles were labeled with FITC to enable characterization of the putative vesicles-on-brush system via fluorescence microscopy. FITC labeling was achieved in aqueous media. Two fluorescein labels were targeted per copolymer chain under mild conditions, which has a negligible impact on the copolymer molecular weight (see GPC data in Figure S4) and vesicle dimensions (see DLS data in Figure S4). The FITC reagent reacted with pendent *cis*-diol groups within the GO_19_ steric stabilizer chains and excess FITC was removed by multiple centrifugation/redispersion cycles, as confirmed by subsequent UV GPC analysis (see Figure S4). FITC-labeled GO_19_-H_500_ vesicles were imaged using fluorescence microscopy prior to adsorption studies. Although individual vesicles were too small to be resolved, strong fluorescence was observed for a 1.0% w/w aqueous dispersion (Figure 4A).

In a control experiment, no fluorescence was observed for a bare PAGEO5MA brush (Figure S5A). Chemical adsorption of the FITC-labeled vesicles was achieved by simply immersing the patterned brush-coated silicon wafers into a 1.0% w/w aqueous dispersion of the FITC-labeled GO_19_H_500_ vesicles at 22 °C for 16 h at pH 5.5 (Figure 4B). Non-specific adsorption of these vesicles to the underlying silicon wafer resulted in a weakly fluorescent background signal. However, a much stronger fluorescence signal was observed for the ∼3 μm patterned lines from which a 109 nm aldehyde-functionalized PAGEO5MA brush had been grown. Thus this experiment confirms the successful preparation of the desired vesicles-on-brush system at pH 5.5. A similar vesicle adsorption experiment was performed at pH 10 (Figure S4B) with no fluorescence being detected in this case. In summary, these observations (and literature precedent) indicate that the chemical adsorption of hydroxy-functional diblock copolymer vesicles at the surface of an aldehyde-functional polymer brush proceeds via acid-catalyzed acetal bond formation.^39,53^ It is also worth emphasizing that the vesicles are not simply grafted to the terminal repeat unit on each brush chain. Instead, acetal bond formation most likely involves the reaction of multiple repeat units located within the upper surface of the brush.^70^

**Figure 5:**
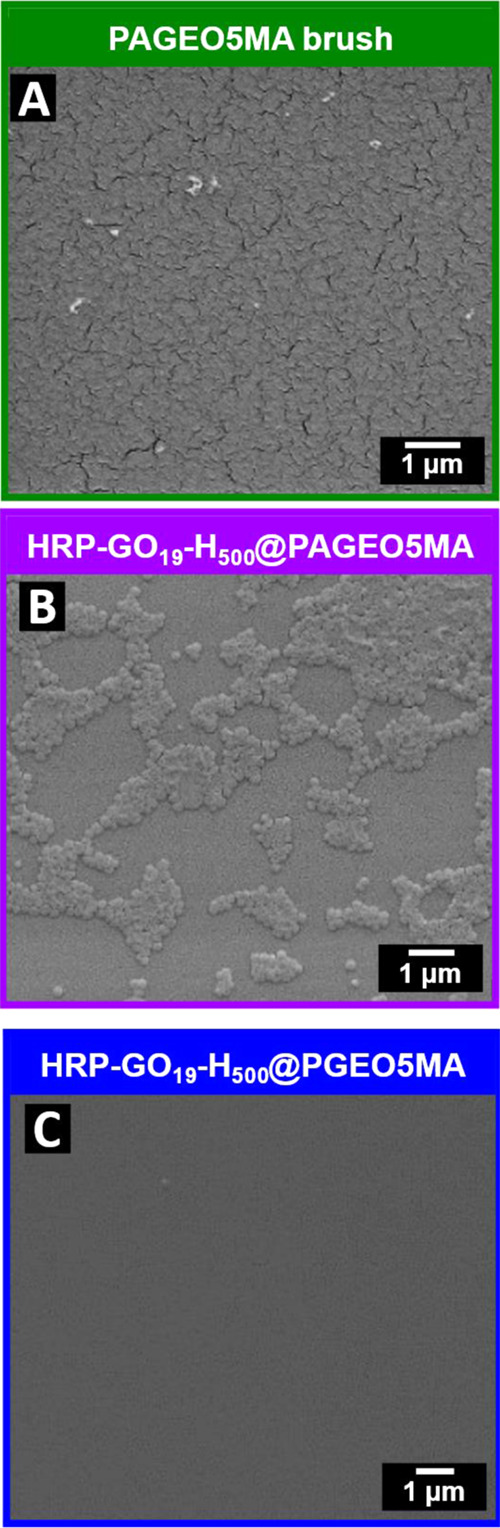
(A) Representative SEM images recorded for PAGEO5MA brush (A) prior to vesicle adsorption and (B) after chemical adsorption via acetal chemistry. (C) SEM image recorded for a non-reactive *cis*-diol-functionalized PGEO5MA brush after exposure to HRP-loaded GO_19_-H_500_ vesicles (control experiment).

SEM analysis of the unpatterned HRP-loaded vesicles-on-brush system confirmed the presence of adsorbed vesicles (compare Figure 5A with 5B). Digital image analysis of such SEM images using ImageJ software indicated a surface coverage of approximately 30%. This is comparable to that observed for the adsorption of aldehyde-functional spherical nanoparticles on brushes via dynamic covalent chemistry.^40^ Furthermore, the surface topography of the dried vesicles-on-brush system was analyzed using AFM (Figure S6). Intact vesicles were observed at the brush surface, and their dimensions were comparable to that of the free vesicles (Figure 2). A control experiment was also conducted to demonstrate that vesicle adsorption involves acetal bond formation, rather than merely physical adsorption onto the brush surface. Accordingly, HRP-loaded GO_19_-H_500_ vesicles were exposed to a PGEO5MA precursor brush prior to its NaIO_4_ treatment. In this case, SEM studies indicated negligible nanoparticle adsorption onto this *cis*-diol-functional brush (Figure 5C). This confirms that the presence of aldehyde groups is essential to facilitate the chemical adsorption of vesicles at the brush surface via acetal bond formation under mildly acidic conditions.

Ellipsometry data obtained for the dried HRP-loaded vesicles-on-brush system were satisfactorily fitted using a single Cauchy parameter. This analysis indicated a mean increase in the dry brush thickness of 23 nm after vesicle adsorption. Bearing in mind the fractional surface coverage of approximately 30% indicated by SEM studies and the mean vesicle membrane thickness of approximately 31 nm estimated from cryo-TEM studies, this suggests that the dried vesicles collapse to form a planar bilayer on the upper brush surface. Similar observations have been reported for lipid adsorption on polymer brushes.^71^

However, the SEM and AFM images shown in Figures 4B and S6C provide no evidence for vesicle rupture. On the other hand, full collapse of the adsorbed vesicles during drying should produce an increase in the mean brush layer thickness of approximately twice the membrane thickness (i.e., 62 nm) if full surface coverage had been achieved. Hence the ellipsometry data suggest a mean surface coverage of (23–62) × 100% = 37%, which is in reasonable agreement with that estimated by digital image analysis of the SEM images.

Ellipsometry studies of the solvated HRP-loaded vesicles-on-brush system indicated an increase in the mean hydrated brush thickness from 190 to 320 nm. In this case, a model comprising two hydrated polymer layers was required to provide a satisfactory fit to the ellipsometric data. To a first approximation, these two layers represent the underlying brush and the adsorbed vesicles, respectively; they are defined by EMAs that differ only in the water volume fraction and the mean layer thickness. However, the vesicle/brush boundary may not be well-defined, so only the combined layer thickness is considered here. The substantial increase in overall thickness noted above is consistent with strong vesicle adsorption. If it is assumed that both the vesicle diameter and the mean brush layer thickness remain unchanged after adsorption, then the observed increase in layer thickness of 130 nm suggests a vesicle surface coverage of around 45%. Clearly, this value lies closer to that indicated by ellipsometry measurements on the dry brush than that suggested by SEM analysis. Given that the ellipsometry data were acquired over a macroscopic area that is a substantial fraction of the entire substrate, the surface coverage indicated by SEM analysis is likely to be an underestimate owing to inferior statistics.

**Figure 6:**
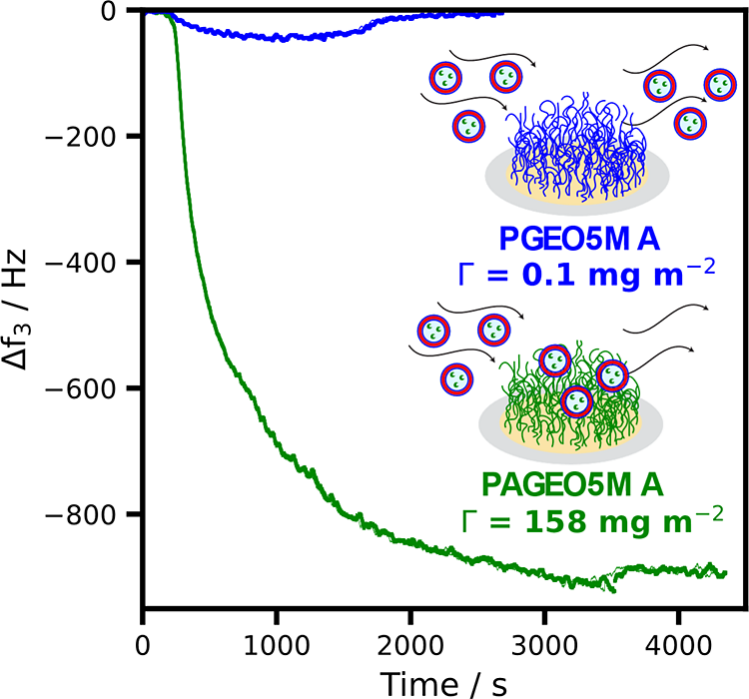
Change in frequency of the third overtone, Δ*f*_3_, over time at 25 °C for a QCM silica sensor coated with either a 43 nm *cis*-diol-functionalized PGEO5MA brush (blue) or a 41 nm aldehyde-functionalized PAGEO5MA brush (green) after exposure to an aqueous dispersion of HRP-loaded vesicles (1.0 wt %) in PB solution at pH 5.5. Using the Sauerbrey equation, the corresponding adsorbed amount, Γ, was calculated to be 0.1 mg m^–2^ for the *cis*-diol-functionalized PGEO5MA brush and 158 mg m^–2^ for the aldehyde-functionalized PAGEO5MA brush.

QCM was used to monitor the mass of adsorbed HRP-loaded vesicles onto PAGEO5MA brushes (Figure 6). QCM monitors the change in the resonant frequency of a quartz sensor and is sensitive to any change in its coupled mass. Brush-coated sensors were prepared using an identical protocol as that employed for planar silicon wafers and then mounted into the QCM instrument prior to exposure to the buffer solution. A change in the frequency for the third overtone, Δ*f*_3_, was monitored as PAGEO5MA, and PGEO5MA brushes were exposed in turn to HRP-loaded vesicles. The adsorbed amount, Γ, was calculated from the final change in frequency and indicates strong adsorption of vesicles onto the aldehyde-functional PAGEO5MA brushes (Γ = 158 mg m^–2^). Conversely, the PGEO5MA brush exhibits antifouling behavior (i.e., minimal vesicle adsorption), which is consistent with the SEM image shown in Figure 5C and our prior study of BSA adsorption.^37^ It is worth noting that the Sauerbrey equation assumes a rigid film, and its use here does not account for the associated water of the hydrated polymer vesicles. Nevertheless, there is clearly a substantial difference in adsorbed mass when exposing the HRP-loaded vesicles to the *cis*-diol- and aldehyde-functional brushes, respectively, which illustrates that the presence of aldehyde groups promotes vesicle adsorption.

### Assessment of Enzymatic Activity for HRP-Loaded Vesicles-On-Brush

**Figure 7:**
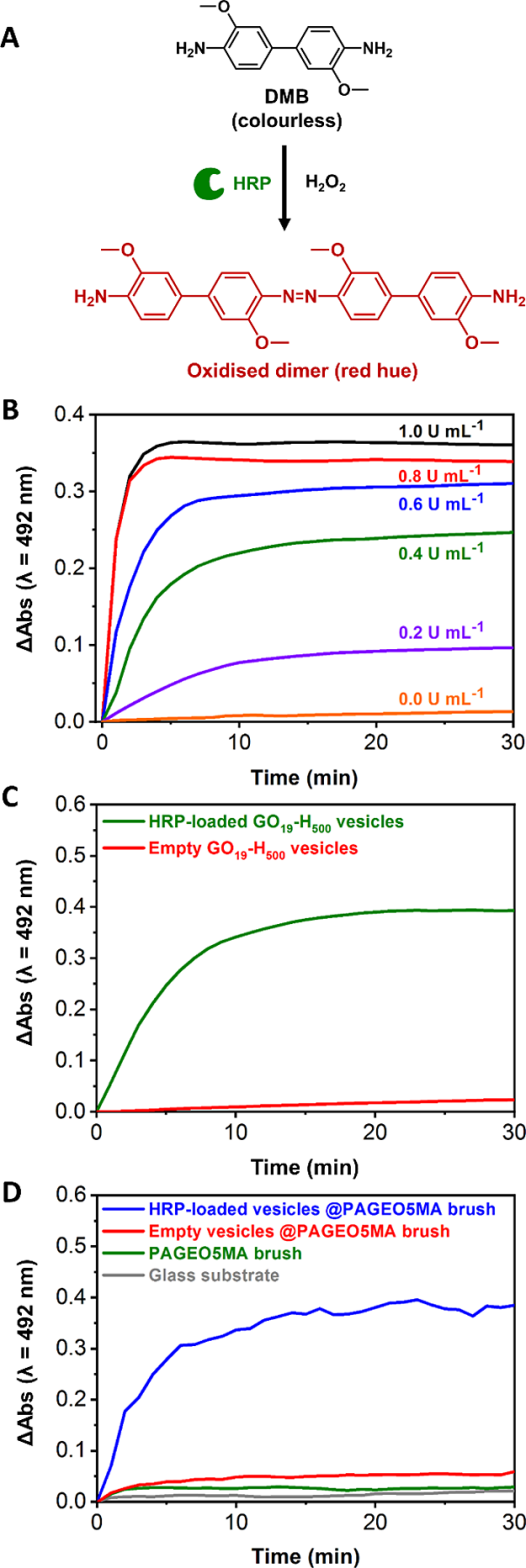
(A) HRP-catalyzed oxidation of DMB generates a red dimer product inside HRP-loaded GO_19_-H_500_ vesicles. (B) Enzymatic activity of free HRP diluted in 100 mM PB solution at pH 5.5 at various enzyme concentrations. (C) Enzymatic activity of purified HRP-loaded GO_19_-H_500_ vesicles (green curve) compared to that observed for empty GO_19_-H_500_ vesicles (red curve). (D) Enzymatic activity of HRP-loaded GO_19_-H_500_ vesicles chemically adsorbed onto an aldehyde-functional PAGEO5MA brush (blue curve) relative to that observed for three control experiments (red, green, and black curves).

A well-established colorimetric assay was used to assess the enzymatic activity of HRP after each synthesis step, as shown in Scheme 2.^13,34,35^ HRP catalyzes oxidation of a colorless substrate, 3,3′-dimethoxybenzidine (DMB), to form a red dimer product (λ_max_ = 492 nm), see Figure 7A. Hence HRP activity can be determined by monitoring the absorbance over time. Initially, enzyme activity was assessed for native HRP (i.e., in the absence of any vesicles) at concentrations ranging from zero up to that corresponding to the maximum theoretical vesicle loading efficiency ([HRP] = 1 U mL^–1^). As anticipated, higher HRP concentrations led to stronger absorbance within a given time frame (Figure 7B). This assay was then used to assess the activity of aqueous dispersions of the empty and HRP-loaded vesicles after purification to remove excess non-encapsulated enzyme (see Figure 7C). Notably, HRP encapsulation within the PGEO5MA_19_-PHPMA_500_ vesicles did not lead to any significant reduction in enzyme activity. This suggests that the PHPMA membranes are highly permeable with respect to small molecules (e.g., DMB and its corresponding red dimer) and offer minimal barrier to their diffusion. On the other hand, such membranes are impermeable with respect to the much larger HRP (ca. 40,000 g mol^–1^; particle diameter = 6 nm), which is therefore retained within the vesicles. Similar findings were reported by Blackman et al. for enzyme-loaded PHPMA-based vesicles.^13,14,34^ Moreover, a control experiment conducted using empty vesicles exhibited a much lower (close to background) absorbance.

Finally, the enzyme activity of HRP-loaded vesicles adsorbed onto a PAGEO5MA brush (dry brush thickness = 120 nm) was examined (Figure 7D). In control experiments, the bare glass substrate, the aldehyde-functional PAGEO5MA brush, and the empty PGEO5MA_19_-PHPMA_500_ vesicles adsorbed onto this brush exhibited no significant increase in absorbance at 492 nm over time. In contrast, the HRP-loaded vesicles-on-brush system exhibited enzymatic activity that was comparable to that of the free (non-adsorbed) vesicles in solution. This indicates that the enzyme within the adsorbed vesicles retains its activity after acetal bond formation at pH 5.5 for 16 h at 22 °C. Notably, these mild reaction conditions are close to the optimum conditions required for maximum HRP activity.^13,14,34^ Given that the vesicles should prevent enzyme deactivation with respect to both denaturation and the presence of proteases, such vesicles-on-brush systems are expected to offer new opportunities as immobilized nanoreactors for microfluidic devices and more generally for flow chemistry applications.

## Conclusions

Well-defined horse radish peroxidase-loaded diblock copolymer vesicles prepared via aqueous PISA can be immobilized at the surface of a reactive aldehyde-functional polymer brush. Vesicle adsorption is achieved via acetal bond formation under mild conditions (pH 5.5, 20 °C, 16 h), which ensures that the activity of the encapsulated enzyme is retained. Moreover, a mean vesicle surface coverage of at least 30% is achieved at the brush surface, which indicates reasonably efficient chemical adsorption. Importantly, the immobilized vesicles remain permeable toward small molecules while retaining their enzyme payload. Such enzyme-loaded vesicles proved to be stable under ambient conditions and their enzymatic activity was demonstrated using a well-established colorimetric assay. In principle, enzyme encapsulation within vesicles should prevent enzyme deactivation on exposure to proteases, which are too large to traverse the vesicle membrane. Moreover, this efficient strategy should be applicable to a wide range of enzymes and functional proteins for the design of next-generation surface-immobilized nanoreactors for enzyme-mediated catalysis.

## Supporting Information Available

^1^H NMR spectrum for Bz-CEPA RAFT agent; ^1^H NMR spectra for the GO_19_ precursor, GO_19_-H_500_ vesicles, and HRP-loaded GO_19_-H_500_ vesicles; AFM image for a PGEO5MA brush grown from a planar silicon wafer; and ellipsometry data for a PAGEO5MA brush grown from a planar silicon wafer plus the corresponding HRP-loaded vesicle-decorated PAGEO5MA brush (PDF)
