# Supplementary material for: Capturing Enzyme-Loaded Diblock Copolymer Vesicles Using an Aldehyde-Functionalized Hydrophilic Polymer Brush
Source: Langmuir. 2024 Jun 27;40(27):14086–98. doi: 10.1021/acs.langmuir.4c01561 (PMC11238591; doi:10.1021/acs.langmuir.4c01561)
Supplement: Supplementary file 1 — la4c01561_si_001.pdf [file la4c01561_si_001.pdf]

Supporting Information for:

Capturing Enzyme-Loaded Diblock Copolymer Vesicles Using An Aldehyde-functionalized Hydrophilic Polymer Brush

Georgios Karchilakis[a], Spyridon Varlas[a] , Edwin C. Johnson[a,*], Oleta Norvilaite[a], Matthew A. H. Farmer[a], George Sanderson[b], Graham J. Leggett[a] and Steven P. Armes[a,*]

[a] Dainton Building, Department of Chemistry, The University of Sheffield, Brook Hill, Sheffield, South Yorkshire, S3 7HF, UK.

[b] GEO Specialty Chemicals, Hythe, Southampton, Hampshire SO45 3ZG, UK.

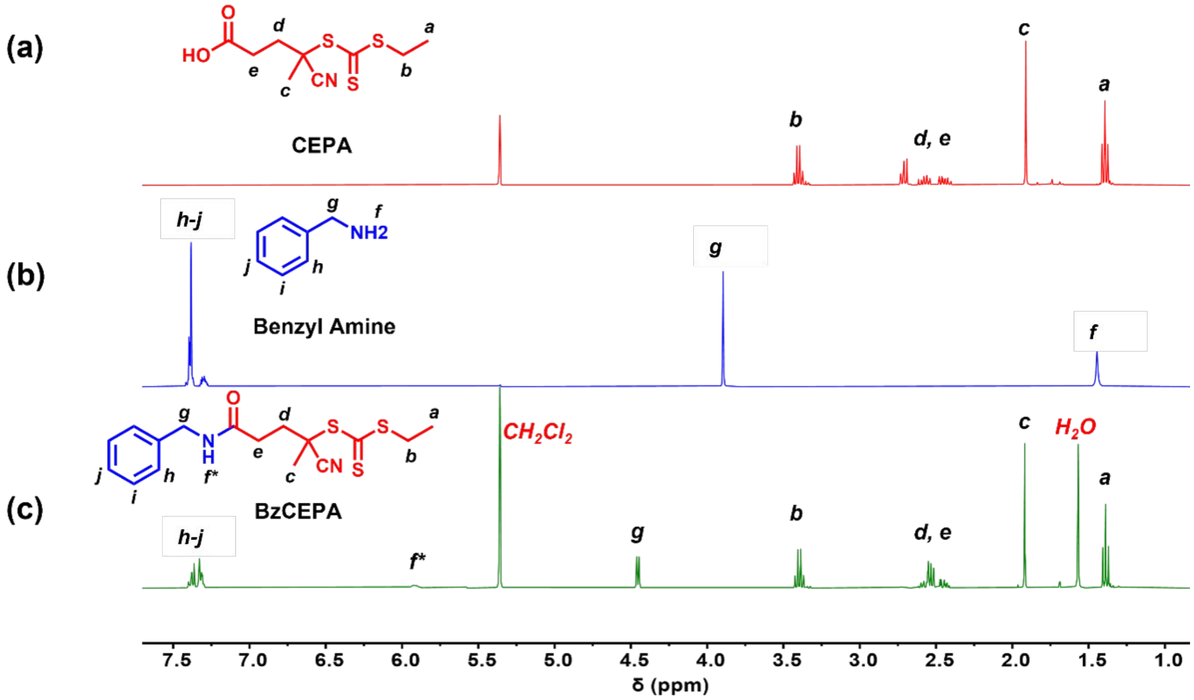

Figure S1. Assigned ^{1}H NMR spectra (CD_2Cl_2) obtained for (a) the carboxylic acid-functionalized RAFT agent (CEPA), (b) benzyl amine and (c) the Bz-CEPA RAFT agent.

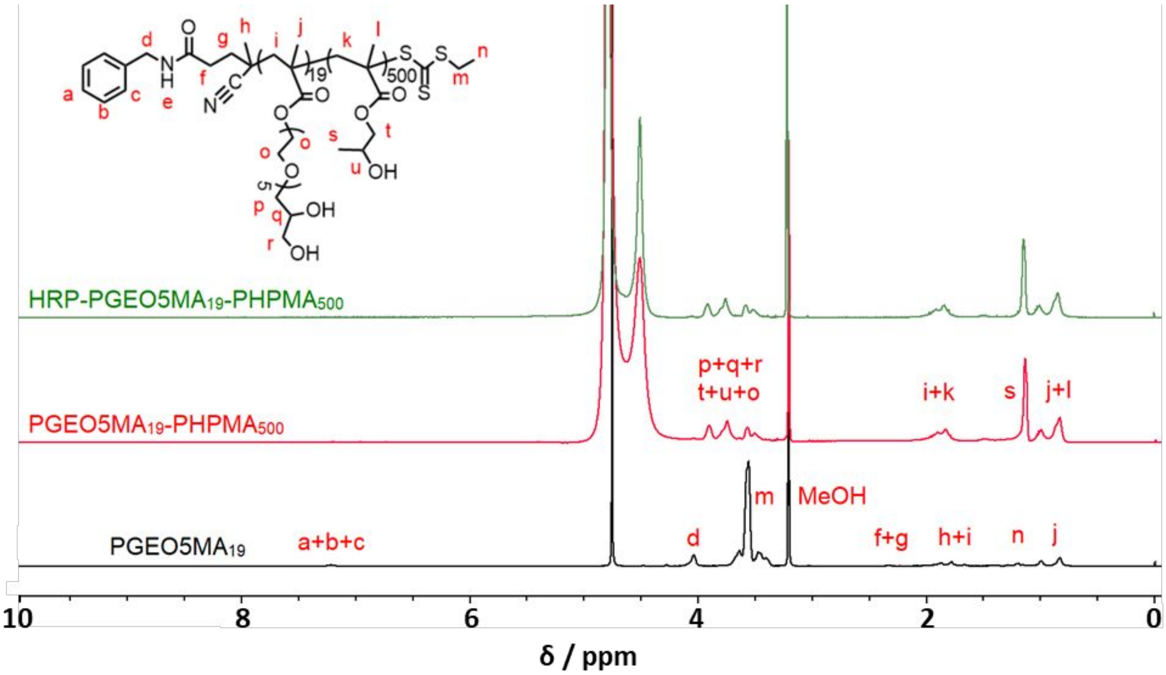

Figure S2. Assigned ^{1}H NMR spectra (CD_3OD) obtained for the purified $PGEOMA_{19}$ precursor (black spectrum), the $PGEOMA_{19}$-$PHPMA_{500}$ vesicles (red spectrum) and the HRP-loaded $PGEOMA_{19}$-H_{500} vesicles (green spectrum). The $PGEOMA_{19}$ precursor was prepared via RAFT solution polymerization of GEO5MA using the Bz-CEPA RAFT agent, while the $PGEOMA_{19}$-$PHPMA_{500}$ vesicles were prepared via RAFT aqueous dispersion polymerization of HPMA in the presence or absence of the HRP enzyme.

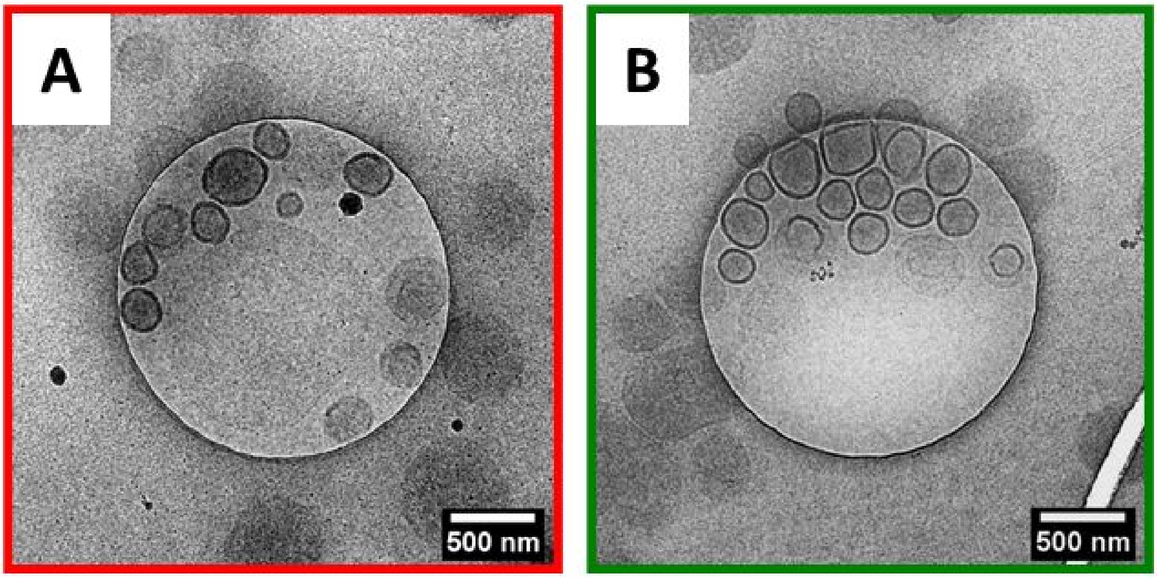

Figure S3. Representative cryo-TEM images recorded for (A) $PGEOMA_{19}$-$PHPMA_{500}$ (GO_{19}-H_{500}) vesicles and (B) HRP-loaded $PGEOMA_{19}$-$PHPMA_{500}$ (GO_{19}-H_{500}) vesicles.

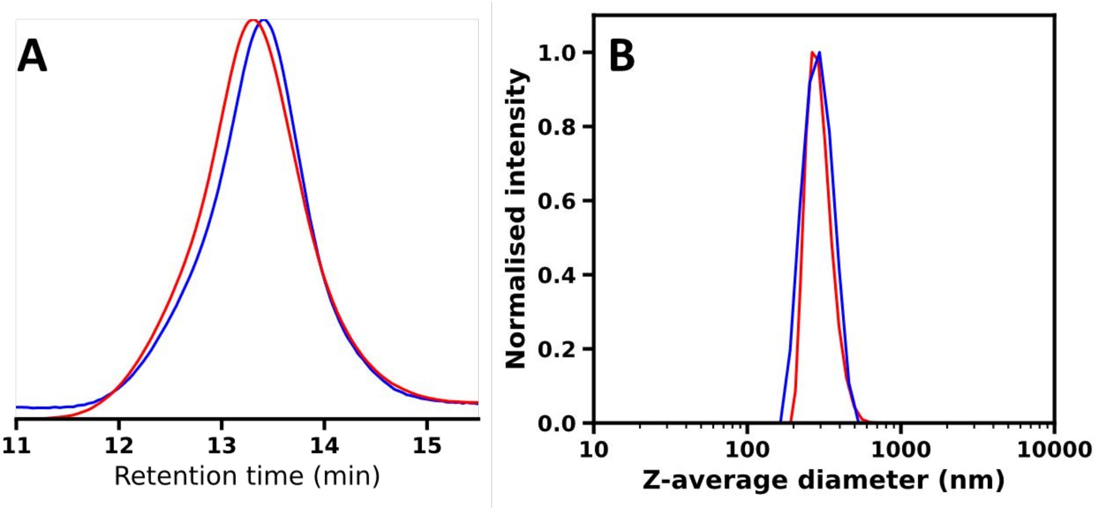

Figure S4. (A) Normalized SEC data recorded for the GO_{19}-H_{500} diblock copolymer (blue curve) and the FITC-$GO_{19}H_{500}$ diblock copolymer (red). (B) DLS particle size distributions recorded for empty (blue curve) and FITC-labelled (red curve) GO_{19}-H_{500} vesicles.

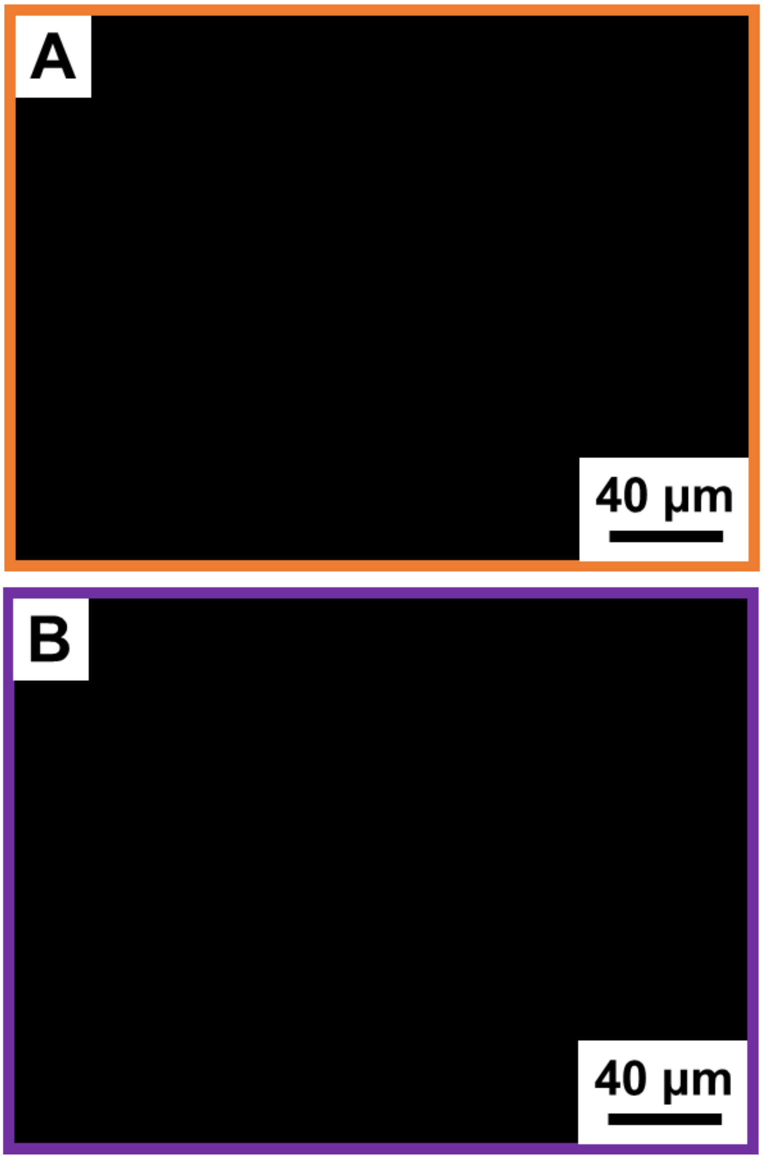

Figure S5. Fluorescence microscopy images recorded for: (A) a bare 116 nm PAGEO5MA brush on glass; (B) attempted adsorption of FITC-labeled $PGEOMA_{19}$-$PHPMA_{500}$ (GO_{19}-H_{500}) vesicles on a 116 nm PAGEO5MA brush at pH 10. No fluorescence was detected for either sample.

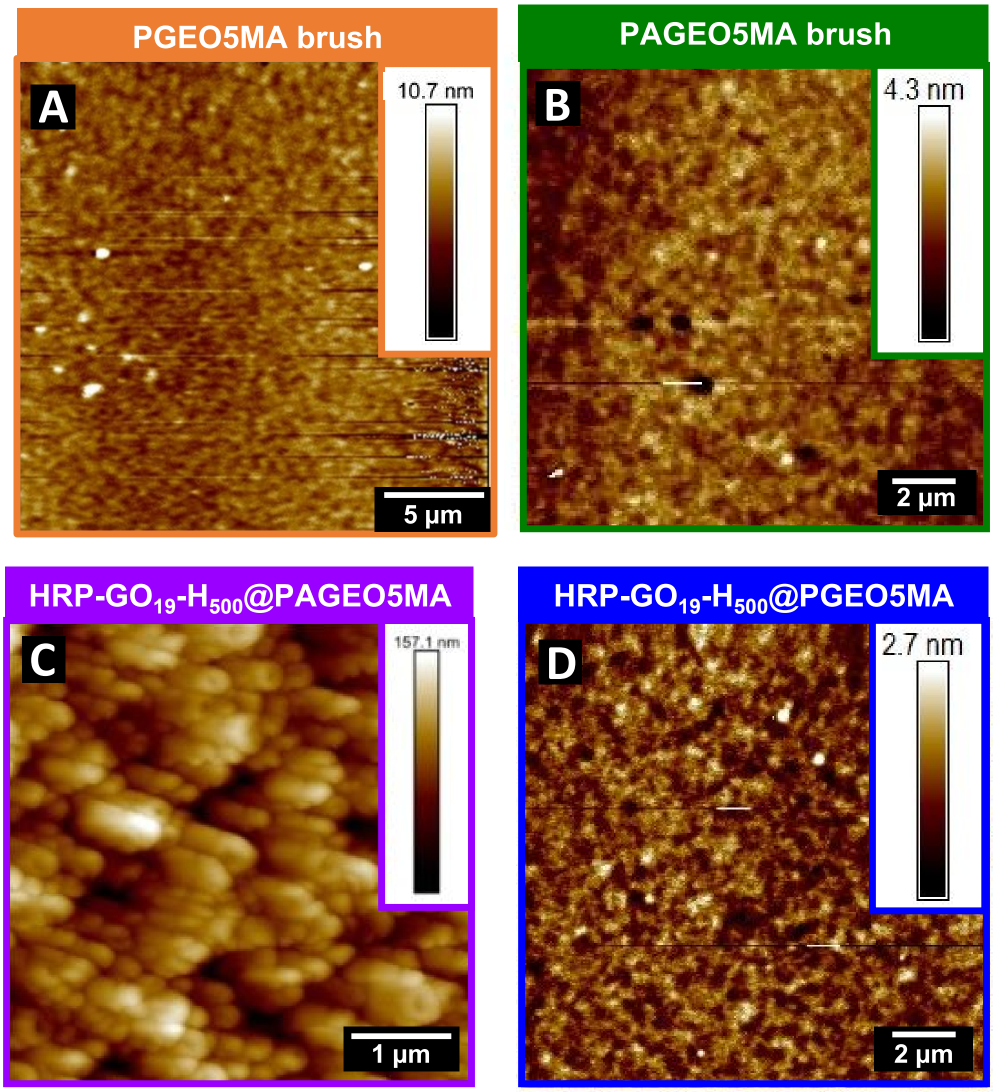

Figure S6. Representative tapping mode AFM images recorded for (A) a PGEO5MA brush, (B) PAGEO5MA brush prior to vesicle adsorption, (C) a PAGEO5MA brush after chemical adsorption of HRP-loaded GO_{19}-H_{500} vesicles, and (D) an unreactive *cis*-diol-functionalized PGEO5MA brush after exposure to HRP-loaded GO_{19}-H_{500} vesicles (control experiment).

Table S1. Summary of ellipsometry data obtained for either dry or wet PAGEO5MA brushes grown from planar silicon wafers, plus the corresponding dry or wet HRP-loaded GO_{19}-H_{500} vesicle-decorated PAGEO5MA brushes.

Entry No.	PAGEO5MA dry brush thickness (nm)	PAGEO5MA wet brush thickness (nm)	Vesicle-decorated PAGEO5MA dry brush thickness (nm)	Vesicle-decorated PAGEO5MA wet brush thickness (nm)
1	120	194	150	316
2	131	200	149	327
3	123	193	141	318
Mean	125	196	147	320
